# Enhancement of orthodontic tooth movement and root resorption in ovariectomized mice

**DOI:** 10.1016/j.jds.2021.11.009

**Published:** 2021-12-02

**Authors:** Yasuhiko Nara, Hideki Kitaura, Aseel Marahleh, Fumitoshi Ohori, Takahiro Noguchi, Adya Pramusita, Ria Kinjo, Jinghan Ma, Kayoko Kanou, Itaru Mizoguchi

**Affiliations:** Division of Orthodontics and Dentofacial Orthopedics, Department of Translational Medicine, Tohoku University Graduate School of Dentistry, Sendai, Japan

**Keywords:** Tooth movement, Orthodontics, Odontoclast, Bone resorption, Root resorption

## Abstract

**Background/purpose:**

As the number of patients with osteoporosis requiring orthodontic treatment is increasing with the aging of society, it is necessary to evaluate the relations between bone metabolism in old age and orthodontic tooth movement (OTM). However, the effects of changes in bone metabolism due to osteoporosis on OTM and root resorption are still unclear. Therefore, we investigated the effects of OTM and root resorption in a mouse ovariectomy (OVX)-induced osteoporosis model.

**Materials and methods:**

Eight-week-old female wild-type mice underwent OVX or sham surgery (Sham) as controls. One month after treatment, a nickel titanium coil spring was used to apply a mesial force to the maxillary left first molars of OVX or Sham mice for 12 days. The distance between the maxillary first molar and the second molar changed due to OTM and osteoclast formation was evaluated. The odontoclast formation and root resorption along the root surface of the distobuccal root of the first molar was also evaluated by histological analysis and scanning electron microscopy.

**Results:**

Distance of tooth movement and osteoclast formation were significantly increased in OVX mice compared to Sham controls. Furthermore, root resorption in the mesial surface of the distal molars induced by orthodontic force was significantly increased in OVX mice.

**Conclusion:**

The amount of OTM was significantly increased, and the accompanying root resorption was also increased in OVX mice. Therefore, attention should be paid to the risk of root resorption associated with orthodontic treatment in patients with osteoporosis.

## Introduction

Osteoporosis is a major disease of postmenopausal women, which is characterized by bone degradation, and is therefore an important public health issue and a topic of significant research interest worldwide.^1^^,^^2^ Postmenopausal osteoporosis is a bone metabolic disease characterized by decreasing bone mass, decreasing bone mineral density, increasing skeletal fragility, and increasing fracture risk due to increased osteoclast bone resorption associated with age-related hormonal changes.^3^, ^4^, ^5^ Current treatments for osteoporosis include denosumab, selective estrogen receptor modulators, parathyroid hormone, teriparatide, bisphosphonates, vitamin D, and calcium.^6^ However, these treatments have various side effects and the therapeutic effects are therefore considered to be less than ideal.^7^ Given the increasing number of orthodontic patients with osteoporosis and other bone metabolic diseases with the aging of society, it is necessary to evaluate the relations between bone metabolism in old age and orthodontic tooth movement (OTM).

Osteoclasts, which are derived from bone marrow cells, indicate bone resorption. Two significant factors are involved in osteoclast formation, i.e., macrophage colony-stimulating factor (M-CSF) and the receptor activator of necrosis factor κB ligand (RANKL).^8^ TNF-α has also been reported to be essential for osteoclast formation.^9^, ^10^, ^11^

OTM is a dynamic process achieved through bone resorption by osteoclasts on the compression side and bone formation by osteoblasts on the tension side. Odontoclasts, which can resorb the tooth root, appear alongside osteoclasts during OTM. RANKL^12^^,^^13^ and TNF-α^14^^,^^15^ are essential factors for differentiation of osteoclasts and odontoclasts during OTM. Root resorption, which is thought to be caused by odontoclasts as well as bone resorption by osteoclasts, is frequently observed as an unwanted side effect of orthodontic treatment and has yet to be resolved.

Osteoporosis is a bone metabolism disorder characterized by age-related changes in bone metabolism, bone resorption, and destruction of bone microstructure. As the number of patients with osteoporosis and other bone metabolism diseases receiving orthodontic treatment will increase with the aging of society, it is necessary to evaluate the relations between bone metabolism in old age and OTM. However, a number of issues regarding the effects of changes in bone metabolism due to osteoporosis on OTM and root resorption remain unclear. This study was performed to investigate the effects of OTM and root resorption in a mouse ovariectomy (OVX)-induced osteoporosis model.

## Materials and methods

### Experimental animals

Female C57BL6/J mice (12 weeks old, n = 4 per group) were purchased from CLEA Japan (Tokyo, Japan), and maintained in the Tohoku University animal facility on a granular diet (Oriental Yeast, Tokyo, Japan). Animals received humane care and all experimental procedures were performed in accordance with the guidelines for care and use of experimental animals of Tohoku University.

### Orthodontic tooth movement in ovariectomized mice

**Figure 1:**
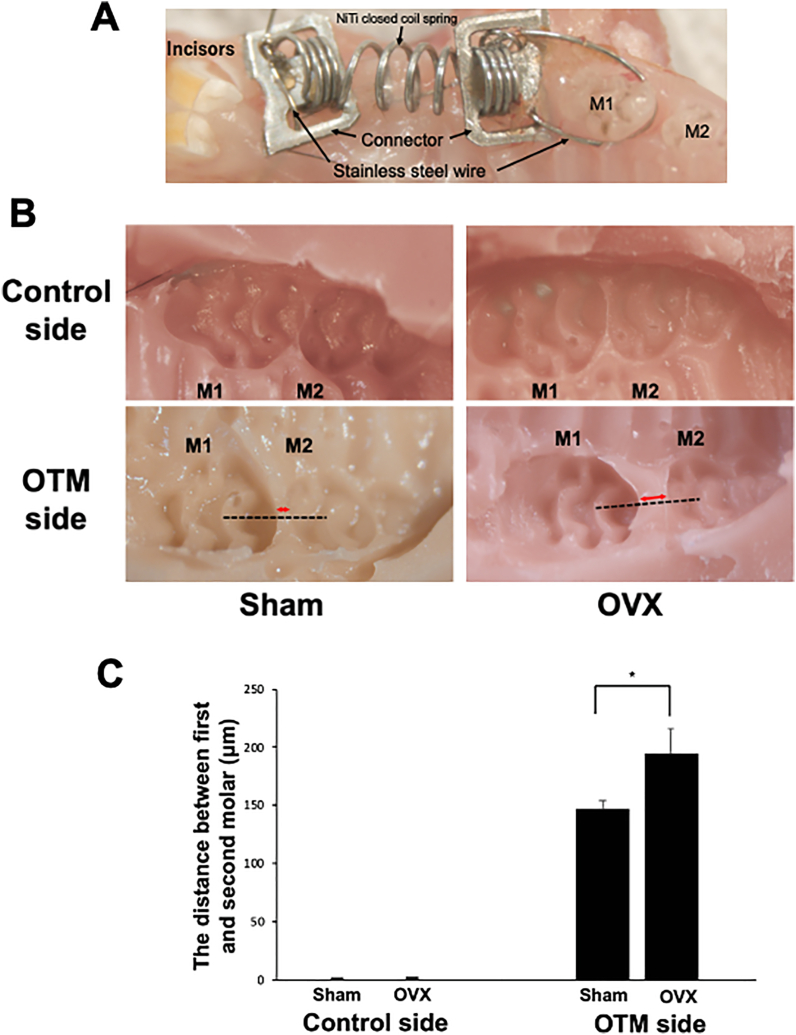
OTM distance on day 12 in the maxillary left first molar area (OTM side) and right first molar area (control side) in Sham and OVX mice. (A) Intraoral photograph of the appliance fixed between anterior alveolar bone and upper left first molar. (B) The distances of tooth movement were measured using silicone impressions of the control and OTM sides in Sham and OVX mice. (C) Comparison of tooth movement between Sham and OVX mice. ∗p < 0.05. n = 4 for each group.

At 30 days after bilateral OVX, both OVX mice (n = 4) and Sham operation (Sham) controls (n = 4) in which the ovaries were raised without resection were anaesthetized. The OVX and Sham mice were weighed to confirm the success of OVX. The OVX mice showed increased body weight in comparison to Sham controls. After anesthesia by intraperitoneal injection of midazolam, butorphanol, and medetomidine into the mice, a nickel-titanium closed coil spring (Tomy, Fukushima, Japan) was attached between the maxillary left first molar and the anterior alveolar bone with stainless steel wire 0.1 mm in diameter (Fig. 1A). The orthodontic appliance was used to move the first molar in the mesial direction as described previously.^16^ In accordance with the manufacturer's instructions, a force of approximately 10 g after activation was applied, and OTM was evaluated after 12 days of force loading.

### Measurement of tooth movement

After 12 days of force loading, the tooth movement space between the first and second molars was determined by taking an impression of the maxillary teeth with hydrophilic vinyl polysiloxane (EXAFAST Injection Type; GC Co., Tokyo, Japan) under deep anesthesia. The distance of tooth movement was measured as the distance between the distal marginal ridge of the left first molar and the mesial marginal ridge of the left second molar as the OTM side, and between the distal marginal ridge of the right first molar and the mesial marginal ridge of the right second molar as control side by stereoscopic micro-scopy (VH-7000; Keyence, Osaka, Japan) as described previously.^17^

### Histological analysis

After 12 days of OTM, the mice were sacrifice and the maxilla was dissected out and fixed overnight in 4% paraformaldehyde at room temperature. After demineralization with 14% ethylene diamine tetra-acetate for 3 weeks at room temperature, the tissues were processed (TP1020 tissue processor; Leica, Wetzlar, Germany), embedded in paraffin, and cut into sections 4 μm thick in the horizontal plane for histological analysis. The distal root of the first molar was evaluated at 100, 140, 180, 220, and 260 μm from the apical surface to the bifurcation surface in each sample. Tartrate-resistant acid phosphatase (TRAP) activity in the sections was analyzed by TRAP staining solution containing naphthol-ASMX-phosphate (Sigma–Aldrich, St. Louis, MO, USA), Fast Red Violet LB Salt (Sigma–Aldrich), and 50 mM sodium tartrate, followed by counterstaining with hematoxylin. TRAP-positive multinucleated cells detected in the lacunae on the surface of the resorbed alveolar bone on microscopic observation were considered to be osteoclasts, while TRAP-positive multinucleated cells located in the lacunae of the resorbed portion of the root surface were considered to be odontoclasts. The number of TRAP-positive cells in the medial part of the distal buccal root of the upper left first molar was evaluated. The mean number of all five sections was calculated and the percentage root resorption area was calculated as the root resorption surface/root surface ratio. ImageJ software (National Institutes of Health, Bethesda, MD, USA) was used to measure the surface area.^18^

### Evaluation of the root resorption area

After 12 days of OTM, the left first molar was carefully extracted. The tissue including the soft tissue surrounding the left first molar was removed in 2% sodium hypochlorite. After the surface of the root was completely cleared, the mesial side of the distobuccal root was observed by scanning electron microscopy (TM-1000; Hitachi, Tokyo, Japan). To standardize specimen orientation, the tooth was rotated distally until it was placed in a visible position, and the mesial side of the distal root was evaluated. The proportion of the resorption area at the mesial side of the distobuccal root was evaluated using ImageJ software (National Institutes of Health) as described previously.^14^

### Statistical analysis

The data were analyzed using Scheffé’s F test. All data are expressed as the mean ± standard deviation. In all analyses, p < 0.05 was taken to indicate statistical significance.

## Results

### Effects of ovariectomy on orthodontic tooth movement

Tooth movement was observed in both OVX and Sham mice. After 12 days of OTM, the distance of tooth movement in OVX mice was 0.195 ± 0.037 mm, while that in Sham controls was 0.147 ± 0.013 mm. There was no significant difference in tooth movement between the OTM side and control side in OVX or Sham mice (Fig. 1B and C).

### Number of osteoclasts along the alveolar bone after orthodontic tooth movement in ovariectomized mice

**Figure 2:**
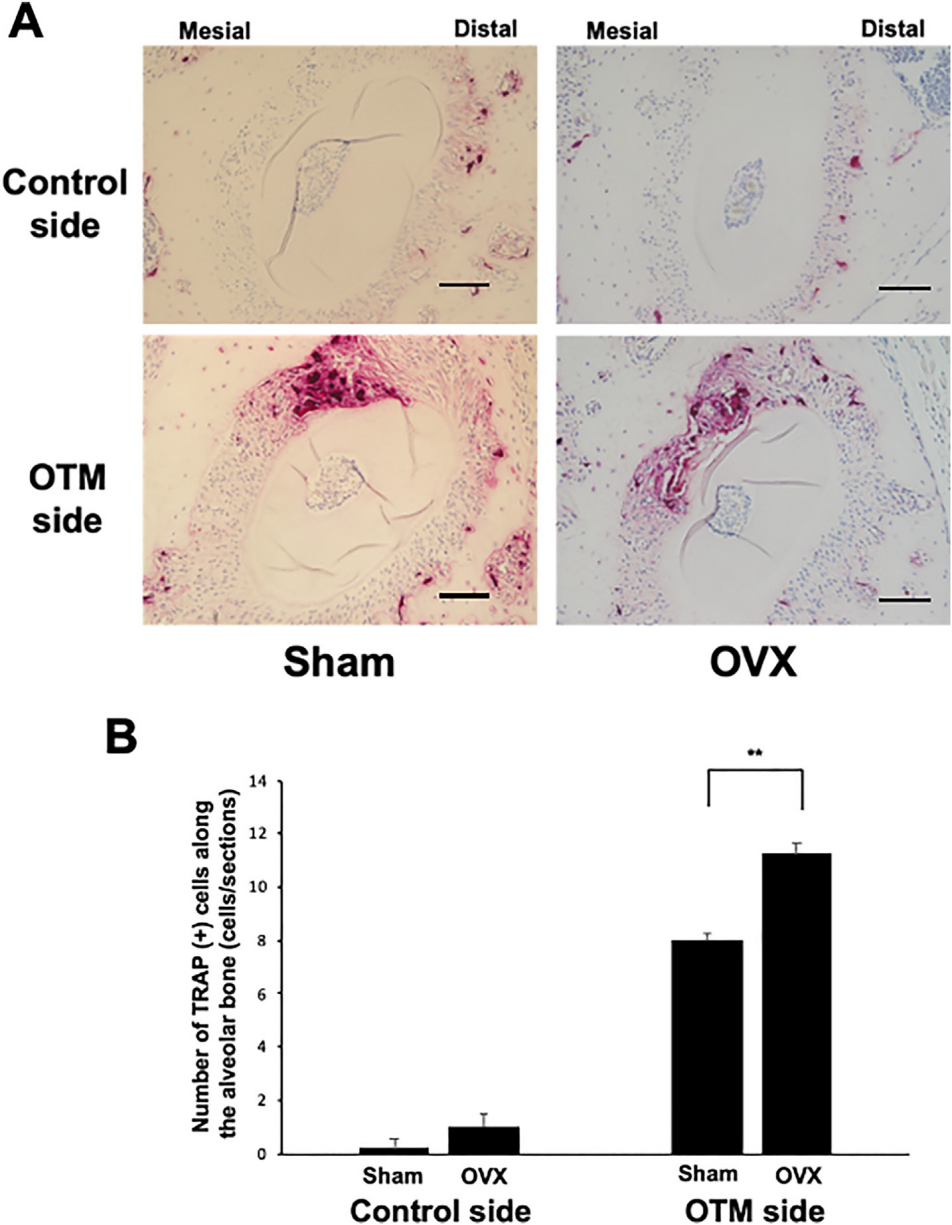
Histological analysis of horizontal sections of alveolar bone in the maxillary left first molar area (OTM side) and right first molar area (control side). (A) TRAP-stained histological sections of the distobuccal root of the maxillary left first molar after 12 days of experimental tooth movement (OTM side) and maxillary right first molar (control side) in Sham and OVX mice. (B) Graph showing the numbers of TRAP-positive cells along the alveolar bone on the pressure (mesial) side in both the maxillary left first molar area (OTM side) and right first molar area (control side) after 12 days in Sham and OVX mice. Scale bars = 50 μm ∗∗p < 0.01. n = 4 for each group.

Tissue sections of the distobuccal root of the first molar on the control and OTM sides were subjected to TRAP staining after 12 days of OTM. The number of TRAP-positive cells along the mechanically loaded alveolar bone was evaluated on the mesial side of the alveolar bone in both OVX mice and Sham controls. The OVX mice showed a significantly increased number of TRAP-positive cells along the alveolar bone (10.75 ± 1.29 cells/section) compared with the Sham mice (8.0 ± 0.71 cells/section) (Fig. 2A and B).

### Effect of ovariectomy on orthodontic tooth movement-induced root resorption

**Figure 3:**
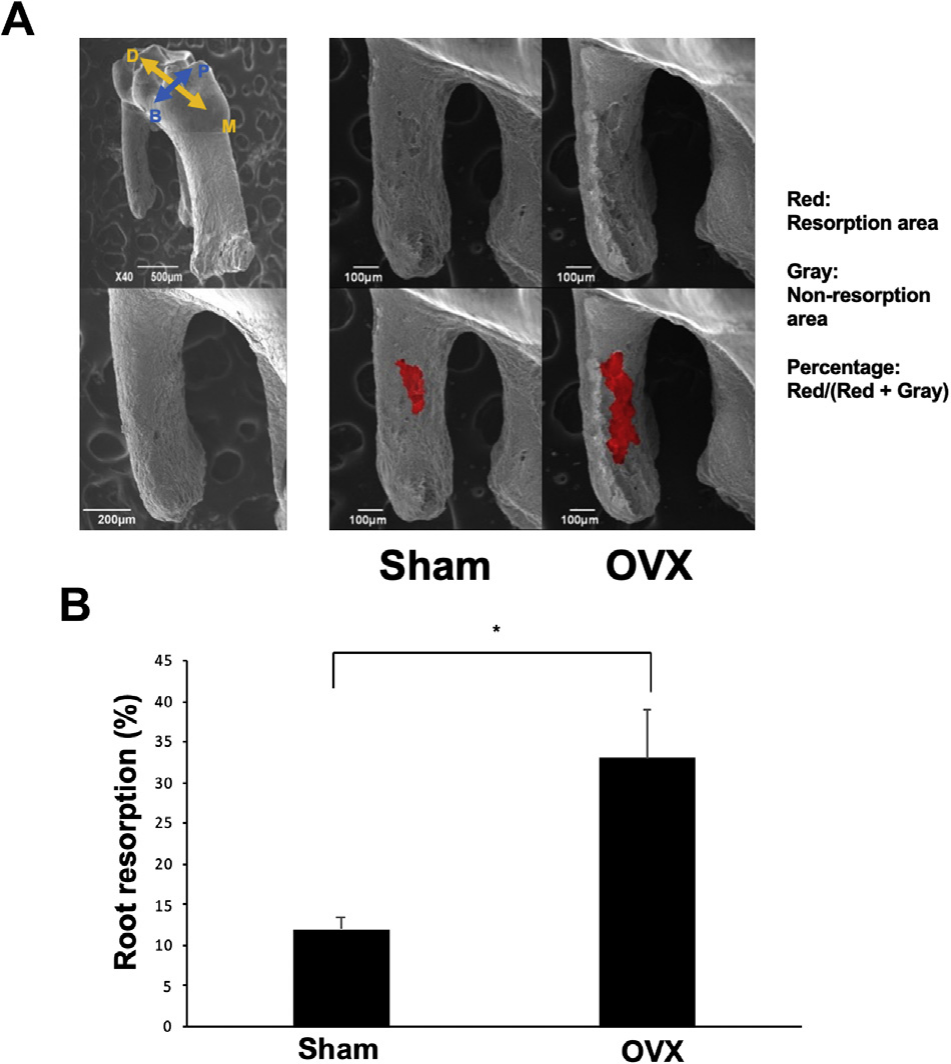
Root resorption after OTM in Sham and OVX mice. (A) Representative scanning electron micrographs of the distobuccal root of the maxillary left first molar after 12 days in Sham and OVX mice. Arrows indicate the orientation of the image: B, buccal; P, palatal; M, mesial; D, distal. (B) Graph indicating the ratio of root resorption area in Sham and OVX mice. ∗p < 0.05. n = 4 for each group.

Root resorption on the mesial side of the distobuccal root was evaluated by scanning electron microscopy. After 12 days of OTM, the area of root resorption along the mesial side of the distobuccal root was significantly increased in OVX mice compared to Sham controls (32.9% ± 14.59% vs. 12.0% ± 5.3%, p < 0.05) (Fig. 3A and B).

### Histological evaluation of orthodontic tooth movement-induced root resorption in ovariectomized mice

**Figure 4:**
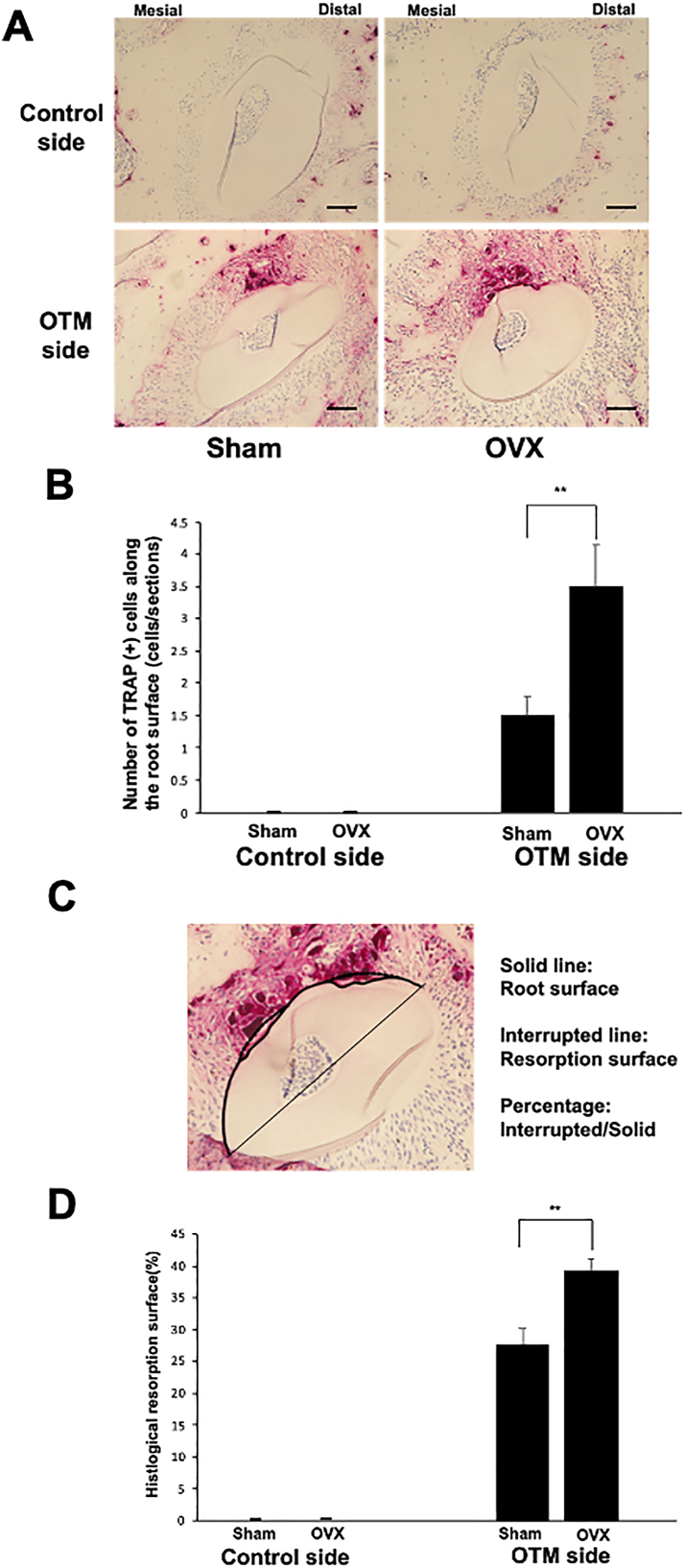
Evaluation of root resorption on transverse histological sections in Sham and OVX mice. (A) TRAP-stained histological sections of the distobuccal root of the maxillary left first molar area (OTM side) and right first molar area (control side) in Sham and OVX mice. (B) The numbers of TRAP-positive cells along the root surface on the pressure (mesial) side of the maxillary left first molar area (OTM side) and right first molar area (control side) after 12 days of OTM in Sham and OVX mice. (C) Evaluation of the root resorption surface on transverse histological sections. The percentage root resorption area was calculated as the root resorption surface (broken line)/root surface (solid line) ratio. (D) Ratio of root resorption surface on histological sections of the maxillary left first molar area (OTM side) and right first molar area (control side) after 12 days of OTM in Sham and OVX mice. Scale bars = 50 μm ∗p < 0.05 and ∗∗p < 0.01. n = 4 for each group.

Odontoclast formation was evaluated after 12 days of OTM. Odontoclasts were detected on the mesial root surface of the distobuccal root of Sham mice (1.5 ± 1.11 cells/section) and showed a significant increase in number in OVX mice (3.5 ± 0.5 cells/section) (p < 0.05) (Fig. 4A and B). Furthermore, the area of root resorption was evaluated on histological sections by stereomicroscopy. On transverse histological sections, the area of root resorption was significantly smaller in the Sham mice than in OVX mice (27.6% ± 3.06% and 39.29% ± 4.42%, respectively, p < 0.05) (Fig. 4C and D).

## Discussion

Given the increasing number of orthodontic patients with osteoporosis and other bone metabolic diseases with the aging of society, it is necessary to evaluate the relations between bone metabolism in old age and OTM. However, the effects of changes in bone metabolism due to osteoporosis on OTM and root resorption are still unclear. In this study, we evaluated the effects of OVX-induced osteoporosis on OTM and root resorption in a mouse model. One month after OVX, a nickel-titanium coil spring was used to exert a mesial force on the maxillary first molars of mice in the OVX and Sham groups. The distance of OTM, osteoclast formation, and root resorption on the medial surface of distal molars induced by orthodontic force was significantly increased in OVX mice compared to Sham controls.

Estrogen plays a crucial role in the microarchitecture of alveolar bone.^19^^,^^20^ Several studies showed that the distance of OTM was increased in OVX rats.^21^, ^22^, ^23^, ^24^ In this study, the distance of tooth movement in OVX mice was greater than that in Sham mice. OVX also enhanced tooth movement in mice. Thus, the OTM in this study showed a similar pattern to previous studies.

OTM is dependent on alveolar bone remodeling by compression force-induced osteoclasts and tension force-induced osteoblasts, which result in bone resorption and bone formation, respectively.^25^ Previous studies showed that OVX enhanced osteoclast formation on the pressure side during OTM in rats.^21^^,^^22^ In this study, osteoclast formation was enhanced in OVX mice during OTM. The results suggested that OVX-induced osteoclast formation and bone resorption resulted in enhanced OTM.

Root resorption is an unavoidable iatrogenic sequela of orthodontic treatment.^26^ The root resorption mediated by odontoclasts has a general morphology similar to that of osteoclast. The cellular mechanisms underlying bone resorption are thought to be very similar to those of root resorption in OTM.^27^, ^28^, ^29^, ^30^ Several studies demonstrated the induction of root resorption in OVX rats and mice.^23^^,^^31^ In the present study, the number of TRAP-positive cells located on the root surface was increased in OVX mice. This study suggested that odontoclast formation may be affected by hormones, such as estrogen, and thus estrogen may play a therapeutic role in controlling OTM and preventing root resorption associated with orthodontic treatment.

The results of the present study indicated that amount of tooth movement was significantly increased in OVX mice as a model of osteoporosis and OTM-associated root resorption was also increased in OVX mice. These observations suggest that changes in bone metabolism in patients with osteoporosis can increase tooth movement during orthodontic treatment, but exacerbate root resorption, and therefore the risk of root resorption associated with orthodontic treatment in osteoporotic patients should be taken into consideration.

## Declaration of competing interest

The authors have no conflicts of interest relevant to this article.
